# 

**Published:** 1907-09

**Authors:** 


THE THIRTY-THIRD ANNUAL MEETING of the Mississippi Valley Medical Association will be held at Columbus, Ohio, October 8, 9 and 10, 1907, under the presidency of Dr. H. Horace Grant, of Louisville, Ky. The orator in Medicine will be Dr. Geo. F. Butler, of Chicago, Ill., and the orator in Surgery will be Dr. Frank D. Smythe, of Memphis, Tenn.

The Association is doing commendable work in furthering the cause of medical research, by offering a prize of $100 for the best original essay upon some medical or surgical topic. The committee of the Association to decide upon this contest is composed of Drs. Hugh T. Patrick, of Chicago; C. H. Hughes, of St. Louis, and A. H. Cordier, of Kansas City.

Preparations are being made on an extensive scale for the entertainment of members and guests by the profession of Columbus, with the following Committee of Arrangements: Chairman, F. F. Lawrence; Secretary, Chas. J. Shepard; Treasurer, William E. Davis; Committee Ways and Means, J. W. Clemmer; Entertainment, J. U. Barnhill; Transportation, W. J. Means; Exhibits, W. J. Means; Reception, J. H. J. Upham; Press and Information, Frank Winders; Halls and Meetings, Earl Gilliam; Badges, J. E. Brown; Registration, Wells Teachnor; Ladies, Mrs. W. D. Hamilton.

